# Violence against Healthcare Workers during the COVID-19 Pandemic: A Review of Incidents from a Lower-Middle-Income Country

**DOI:** 10.5334/aogh.3203

**Published:** 2021-04-23

**Authors:** Omaima Anis Bhatti, Hareem Rauf, Namrah Aziz, Russell Seth Martins, Javaid A. Khan

**Affiliations:** 1 Medical College, Aga Khan University Hospital, Stadium Road, Karachi, 74800, Pakistan; 2 Section of Pulmonary and Critical Care Medicine, Department of Medicine, Aga Khan University Hospital, Stadium Road, Karachi, 74800, Pakistan

## Abstract

**Background:**

Healthcare workers (HCWs) across the globe have met tremendous challenges during the COVID-19 (coronavirus disease 2019) pandemic, such as shortages of personal protective equipment, extensive work hours, and constant fear of catching the virus or transmitting it to loved ones. Adding on to the already existing burnout, an increase in incidents of violence and aggression against HCWs was seen in Pakistan and globally.

**Objectives:**

Primarily to review cases of violence against HCWs in Pakistan, highlighting and comparing the instigating factors seen within the country and globally. Secondly, to enlist possible interventions to counter workplace violence in healthcare during a pandemic and in general.

**Methods:**

Incidents of violence towards HCWs in Pakistan during the COVID-19 pandemic occurring between April 7, 2020, and August 7, 2020, were included. The incidents reported from local newspapers were reviewed.

**Findings and Conclusion:**

A total of 29 incidents were identified, with perpetrators of violence most commonly being relatives of COVID-19 patients. Most frequent reasons included mistrust in HCWs, belief in conspiracy theories, hospitals’ refusal to admit COVID-19 patients due to limited space, COVID-19 hospital policies, and the death of the COVID-19 patients. Protests by doctors and other HCWs for provision of adequate PPE, better quarantine conditions for doctors with suspected COVID-19, and better compensation for doctors on COVID-19 patient duty resulted in police violence towards HCWs. To avoid such incidents in the future, institutions, healthcare policymakers, media organisations, and law enforcement agencies must work together for widespread public awareness to counter misconceptions and to exhibit responsible journalism. In hospitals, measures such as de-escalation training and increased security must be implemented. Furthermore, law enforcement agencies must be trained in non-violent methods of crowd dispersal and control to manage peaceful protests by HCWs over legitimate issues.

## Introduction

With the COVID-19 (coronavirus disease 2019) pandemic having caused more than 58 million reported cases and 1 million reported deaths globally as of November 20, 2020, healthcare workers (HCWs) are facing unprecedented challenges while remaining on the frontlines [1]. These include exhaustive work hours, shortages of personal protective equipment (PPE), and a constant fear of contracting COVID-19 themselves or transmitting it to their loved ones. To add to these challenges, a worrying surge in violence against healthcare workers has been reported globally [2,3]. According to the World Health Organization (WHO), up to 38% of HCWs encounter physical violence at some point in their careers, which causes psychological distress and burnout and in turn affects healthcare delivery [4]. In Pakistan, one third of all HCWs have reported aggression directed towards them, with the most common being in verbal form [5]. As the number of confirmed COVID-19 cases in Pakistan crosses 374,000 [6], growing tensions amongst the population have led to an increase in incidents of violence and hostility all over the country. In this review, we describe the incidents of aggression and violence towards HCWs during the COVID-19 pandemic in Pakistan, as reported by local newspapers. In addition, factors motivating these incidents are discussed and related solutions are suggested to provide healthcare systems and the Government of Pakistan with evidence-based action plans to negate such events in the future.

## Search Strategy and Selection Criteria

In the absence of any published literature, our search strategy consisted of reviewing reports of violence against healthcare workers by local news agencies in Pakistan, which uploaded their reports/articles to their websites from April 7, 2020, to August 7, 2020. News websites searched included The Express Tribune, Geo News, The Nation, Dawn News, Samaa News, The News International, News Intervention, Gandhara, National Public Radio (NPR), and CNN. The search was independently performed by two members of the research team (NA and HR), who compiled lists of relevant articles on MS Excel. Once the search was completed, a third member of the research team (OAB) reviewed and synthesized both lists. Duplicates (reports of the same incident in different news reports) were excluded. Discrepancies in lists were settled by referring to the news article in question and determining its suitability for inclusion.

## Violence against HCWs during the COVID-19 Pandemic in Pakistan

A total of 29 incidents were identified through review of local newspaper reports from April 7, 2020, to August 7, 2020 [7,8,9,10,11,12,13,14,15,16,17,18]. The majority of reported incidents took place in the province of Khyber Pakhtunkhwa (69%) [12,15,16], followed by Sindh (13.8%) [9,11,13,14], and Punjab (10.3%) [8,10,17]. All hospitals involved in these incidents of violence were public hospitals owned by the provincial/federal governments [7,9,10,11,12,13,14,15,17]. Perpetrators of the violence were most commonly mobs comprising attendants of patients (60%) [9,10,11,12,13,15] and members of the police/armed forces (40%) [7,8,14,18], and all reported incidents had some degree of physical assault and violence. In the incidents where the police perpetrated violence, the inciting factors were mostly protestations by doctors and other HCWs [7,8,18]. The demands included provision of adequate PPE, better quarantine conditions for doctors with suspected COVID-19, better compensation for doctors on COVID-19 patient duty, and the provision of internet services to facilitate online medical education during the COVID-19 pandemic [7,8,18]. In all these incidents, the police physically assaulted doctors and healthcare workers and even arrested protesters in some cases [7,18]. In incidents perpetrated by mobs led by patients’ attendants, reasons most frequently included healthcare authorities’ refusal to admit COVID-19 patients due to limited space, the death of relatives in the hospital, and the refusal to hand over deceased patients’ bodies without the results of a COVID-19 test [9,10,11,12,13,15,19]. Attendants commonly displayed distrust in doctors, accusing them of falsely diagnosing patients with COVID-19 or providing inadequate healthcare to COVID-19 patients [11,12,13]. Moreover, false beliefs, such as COVID-19 being a hoax and doctors intentionally killing patients while labelling them as COVID-19 positive in order to receive money, were also reasons behind some of the incidents [10,13,16,19]. The violence demonstrated by attendants and mobs included destruction of hospital property, verbal abuse, and physical assault against doctors and hospital staff [9,10,11,12,13,15]. In four out of six incidents involving patient attendants inciting violence at hospitals, the hospital administration sought support from the police/law enforcement personnel [9,10,11,13]. A brief summary of all the incidents of violence is shown in ***Table 1***, and additional details are shown in ***Table 2***.

**Table 1:** Summary of Incidents during the COVID-19 Pandemic in Pakistan.


CHARACTERISTIC	N = 29 n (%)

**Province** **Khyber Pakhtunkhwa [12,15,16]** **Sindh [9,11,13,14]** **Punjab [8,10,17]** **Balochistan [7,18]**	20 (69.0) 4 (13.8) 3 (10.3) 2 (6.9)

**Hospital Sector** **Public (Government-owned) [7,9,10,11,12,13,14,15,17]** **Private**	N = 9 * 9 (100) 0 (0)

**Perpetrator** **Attendants of Patients [9,10,11,12,13,15]** **Police/Armed Forces [7,8,14,18]**	N = 10 * 6 (60.0) 4 (40.0)

**Victim** **Doctors [7,8,9,10,11,12,13,14,15,17,18]** **Other HCWs/Hospital Staff [7,9,10,11]** **Medical Students [18]**	N = 11 * 11 (100) 4 (36.4) 1 (9.1)


* Number of articles explicitly reporting specific characteristic.

**Table 2:** Incidents of Violence Against Health Care Workers (HCW’s) During COVID-19 Pandemic in Pakistan (7 April to 24 June 2020).


CASE #	DATE	CITY	HOSPITAL/ LOCATION	HOSPITAL SECTOR	PERPETRATOR(S)	VICTIM(S)	ACT OF VIOLENCE	INCITING FACTOR(S)	LEGAL ACTION TAKEN	OTHER DETAILS

**1** [7]	6 Apr 2020	Quetta (Balochistan)	Civil Hospital/CM secretariat	Government	Police	Doctors and Paramedics	Baton charge; dragged through streets; Arrested between 60-100 HCWs including doctors and paramedics	Protests held by young doctors and paramedic staff for provision of PPE and equipment to treat coronavirus patients.	NS	Protests began after more than 16 doctors in Quetta contracted COVID-19 due to lack of PPE. Doctors face psychological stress and trauma dealing with patients without PPE (‘akin to suicide’)

**2** [8]	18 Apr 2020	Lahore (Punjab)	Office of Secretary Specialized Healthcare and Medical Education	–	Police	Doctors	Police thrashed the doctors	Protests for provision of adequate PPE, compensation for COVID-19 duty, better quarantine conditions for doctors with suspected COVID_19	NS	Authorities claimed to have called police only to maintain order during protests; Special Secretary Health stated that during the COVID-19 pandemic was not the right time for doctors to hold such protests

**3** [9]	15 May 2020	Karachi (Sindh)	Jinnah Postgraduate Medical Centre (JPMC) Isolation Ward	Government	Around 60 people, including attendants of deceased patient (COVID-19 suspected)	Doctors and Hospital Staff	Forceful entry into premises; heavy objects thrown at staff; Hospital area ransacked and vandalised	Hospital administration refused to hand over the patient’s body to the family	Police and Rangers called to facility; arrested eight to nine people	Hospital staff directed by government to only hand over deceased body to family after conducting a COVID-19 test; Attendants refused to believe that coronavirus exists

**4** [10]	20 May 2020	Lahore (Punjab)	Mayo Hospital, COVID-19 Ward	Government	Around 30 men; attendants of 2 COVID-19 positive patients, one of whom had died.	Doctors and Hospital Staff.	Forceful entry into premises. Verbal abuse and misbehaviour; manhandled male doctor and hospital guard.	Death of COVID-19 patient; relatives believed coronavirus is a hoax	Hospital administration requested deployment of sufficient police force	A male doctor was forced by attendants to perform CPR on the patient without giving him time to wear PPE The other patients in the COVID-19 ward also started to cry and get upset.

**5** [13]	28 May 2020	Hyderabad (Sindh)	Liaqat University Hospital	Government	Attendants of deceased COVID-19 patient	Doctors	Misbehaviour	Refused to hand over body of deceased patient to family; Doctors falsely accused of labelling patients as COVID-19 positive; mistrust in doctors	Hospital administration sought police support	–

**6** [11]	29 May 2020	Karachi (Sindh)	Civil Hospital, Emergency Department	Government	Around 70 people, including attendants of deceased COVID-19 patient	Doctors and Hospital Staff	Forceful entry into premises; mob was carrying knives and rods; attempted assault on medical staff; female doctor slapped; vandalised the area	Patient was brought in critical condition and died during treatment; attendants refused to wait for completion of legal formalities including COVID-19 testing; broke in to retrieve body	Police had decided to register the case against the persons involved.	Doctors claim confusing policies of government regarding burial of suspected COVID-19 patients is a cause to such increasing incidents

**7** [12]	1 Jun 2020	Peshawar (Khyber Pakhtunkhwa)	Hayatabad Medical Complex	Government	Attendants of a suspected COVID-19 patient	Young male doctor	PPE was forcefully removed; Verbally abused and beaten; Sustained injuries to eyes and nose	Doctor was called to take nasal swab after a duty in the ICU; Attendants claimed he arrived late	Police support sought but first information report was not filed yet	Fourth such incident in the last seven days; doctors demand better security from provincial government

**8** [14]	18 Jun 2020	Karachi (Sindh)	National Institute of Cardiovascular Disease (NICVD)	Government	Counter-Terrorism Department Policeman	Young male doctor	Verbal abuse and threats; gunfire injuring both legs	Was denied sleeping pills day before; quarrelled over being asked to wear a mask	Arrested and FIR lodged; Court sent the perpetrator on a 14-day judicial remand.	Perpetrator has been labelled as mentally unstable

**9** [15]	19 Jun 2020	Peshawar (Khyber Pakhtunkhwa)	Lady Reading Hospital	Government	Attendant of deceased patient; Suspected COVID-19	Doctors	Attempted assault on doctors; Hospital property damaged	COVID-19 test ordered to determine cause of death of the elderly patient brought in critical condition	NS	–

**10–27** [16]	As of 19 Jun 2020	(Khyber Pakhtunkhwa)	–	–	–	–	There have been at least 20 attacks on medical workers; include three shooting incidents	Relatives of COVID-19 patients frustrated over lack of testing facilities and due to conspiracy theories stating doctors are killing patients and claiming they died of COVID-19; hospital refused to hand over body of deceased until COVID-19 test results returned	NS	Some conspiracy theories are made by religious clerics and widely spread on social media 20 total reported cases of violence were reported by the Young Doctors Association (two have been described earlier: Case 7 and Case 9)

**28** [17]	21 Jun 2020	Lahore (Punjab)	Jinnah Hospital	Government	NS	Senior Professor of Medicine	Physically threatened	NS	NS	–

**29** [18]	24 Jun 2020	Quetta (Balochistan)	–	–	Police	Female doctors and medical Students	Baton charge; physically assaulted and arrested	Female doctors and medical students peacefully protesting the decision of the Higher Education Commission to conduct online education in the absence of internet facilities in Balochistan	NS	–


NS: not specified; PPE: personal protective equipment.

## Discussion

More than 400 incidents of violence disrupting healthcare were reported worldwide with over 260 cases of aggression being responses to COVID-19 health measures. According to the International Committee of the Red Cross (ICRC), a total of 611 incidents of violence and harassment took place in the first six months of the pandemic [20]. However, these numbers could be understated, because most cases go unreported [21]. A majority of the targets are doctors and nurses directly dealing with COVID-19 patients, and perpetrators either include the family of COVID-19 patients, the general community, or law enforcement personnel [2].

According to our review of cases in Pakistan, the attacks on health care workers were mainly driven by grievances over the death of COVID-19 patients, a mistrust towards doctors borne of widespread conspiracy theories, and resistance towards protective COVID-19 measures set by the government. Cases of violence and harassment against HCWs with similar inciting factors occurred across the globe. In India, a quarantine facility was vandalised, and HCWs were assaulted over the death of a COVID-19 patient [21]. In Central America and Caribbean countries, doctors and nurses were attacked on multiple occasions due to frustration caused by lack of proper care, concerns regarding COVID-19 testing, and reluctance to adhere to rules regarding burial of COVID-19 patients [22]. In the United States, public health officials have been targets of physical threats, protests, and cyber harassment as a result of opposition to usage of masks and lockdown policies, coupled with criticism from political leaders and social media slander campaigns [23]. An additional factor contributing to violence is the fear of catching the virus from HCWs. Several nurses in Mexico were assaulted while travelling to work, a nurse was attacked with bleach in Philippines, and in India, HCWs were chased by mobs, threatened by neighbours and landlords to vacate their homes, and faced hostility during contact tracing [3,24,25,26]. In countries already affected by conflict, like Libya and Yemen, bombings and shelling on healthcare facilities have severely affected the COVID-19 response. In Afghanistan, a maternity ward was attacked by gunmen in May 2020 [22].

Workplace violence in healthcare has been extensively documented in literature. Several multicentre studies have been conducted in Pakistan, and worldwide, to document the magnitude, types, and various factors involved in violence against the medical profession. In a systematic review published in 2019, Liu and colleagues reported that 61.9% of HCWs experienced some form of violence in the past year, with verbal abuse being the most common, and 24.4% HCWs experienced physical violence [27]. The emergency department is the most common site, and most cases are unreported because HCWs consider these aggressions towards themselves as a norm. In Pakistan, incidents have ranged from bullying, harassment, vandalism, and showing of weapons, to threats of extortion and kidnapping [5]. Workplace violence is more prevalent in public hospitals, as compared to private hospitals, due to overcrowding, easy access to the facility by the public, and resource constraints. A similar trend can be observed during this pandemic as well, because almost no cases of violence have been reported from private hospitals [5]. Major contributing factors to these incidents are unexpected outcomes or death of a patient, unavailability of resources at the facility, long waiting times and delay in treatment, miscommunication with patients and their families, and a general lack of awareness in society. Mostly relatives of the patients instigate the violence, as seen in this pandemic too. In the past, misconceptions regarding polio vaccinations have triggered incidents of violence towards health workers, similar to how misinformation regarding COVID-19 has fuelled attacks recently. Another reason observed in the past is the relatives falsely blaming doctors for harming their patients, and a similar pattern can be noted in the incidents of violence that have taken place in the past months [28].

Violence against HCWs can lead to increased work-related stress, burn out, and post-traumatic stress disorder. It negatively affects job performance, in turn leading to poor quality of health care [5]. It is also an additional contributor to already existing psychological distress caused by COVID-19. HCWs are working extra hours, with inadequate PPE and a shortage of medical equipment like ventilators, all the while being at a higher risk of getting infected and spreading it to their families [3,26]. As of July 2020, over 5,000 healthcare workers had been infected with COVID-19 in Pakistan, and 58 lost their lives [29]. Having an increased risk of exposure to COVID-19, with an addition of violent incidents, HCWs developed increased anxiety and depression, and some were even compelled to quit their duties [30].

The impact and consequences of violence, especially during an ongoing pandemic, warrants a need to implement evidence-based solutions to counter such events in the future. We categorised our proposed solutions obtained from a review of existing literature into a pandemic-oriented approach and general interventions to counter workplace violence in healthcare.

## Pandemic and Healthcare Crisis Oriented Solutions

Widespread misinformation about the disease, coupled with already heightened fear and anxiety within the community, seems to be the most notable driving factor in violence towards HCWs worldwide. Therefore, in a global healthcare crisis it is imperative to effectively educate the public through awareness campaigns early on; provide easily accessible, credible sources of information; and ensure speeches made by political leaders and people of influence are verified with facts. Secondly, scientific basis of health policies, and disease experiences from the people themselves, should be communicated to the public to ensure compliance with protective health measures [2]. Some examples of actions taken to counter misconceptions during this pandemic include ‘Stop the Spread’ campaign by WHO in conjunction with the UK government and removal of unsubstantiated content from social media giants like Facebook and Twitter [31,32]. Even though the spread of the virus seems to be slowing down in our part of the world, the introduction of the COVID-19 vaccine may bring new challenges. In a cross-country study, 8.4% of HCWs reported misconceptions about vaccines being a cause of verbal violence [5]. To prevent future hostilities, our leaders and media will have to exhibit responsible journalism.

In light of a pandemic, local governments need to invest in hospital security measures as part of their response budgets, while hospital administrations need to formulate a standard action plan to be followed during any conflict [3]. Additionally, it is important to extend support and to promote self-care practices for HCWs exposed to high levels of stress [33].

Furthermore, offenders must be held accountable for their actions and prosecuted adequately. Following recent incidents during this pandemic, governments have implemented new policies for protection of HCWs, for example, imprisoning perpetrators for up to seven years with a fine of up to five lakh in India [34], and introducing dedicated transport for HCWs in some cities of Mexico to ensure their safety [35]. Lastly, a comprehensive database of such incidents will help understand the scope of this problem and will help us learn lessons from the past, because violence against HCWs is a repeating trend during pandemics and epidemics [2].

## General Measures to Counter Workplace Violence in Healthcare

Organisationally, establishing aggression management teams for de-escalating and controlling incidents of violence in earlier stages can significantly reduce consequences of workplace violence [36]. A strict zero-tolerance attitude towards threats or any forms of violence against staff members should be enforced, along with proper reporting platforms, counselling avenues for victims, and follow-ups on prosecution of offenders [36,37]. Patient education can decrease incidents of violence by 18.6%; therefore, patients and their families need to be well informed of management plans and possible outcomes. Furthermore, reduction in waiting times and strict one-attendant policies to avoid overcrowding can help decrease frustration amongst patients [23,38].

Training HCWs in de-escalation techniques, as advised by Occupational Safety and Health Administration guidelines [39], along with recognition of perpetrators and self-defence, can ensure their protection. Development of interpersonal skills can prove useful when instructing perpetrators to stop the violent act [40].

Lack of security majorly contributes to escalation of such violent incidents [37]. Security measures include use of metal detectors at entrances, making fewer areas accessible to the public by using door locks and card readers, cautious checking for weapons on people entering the facility, and installing functioning CCTV cameras in high risk areas like emergency departments and waiting rooms. These measures, however, can prove costly, especially in rural areas. The Pakistan government should consider shifting at least 3% of the total GDP towards the healthcare sector [41].

**Figure 1:**
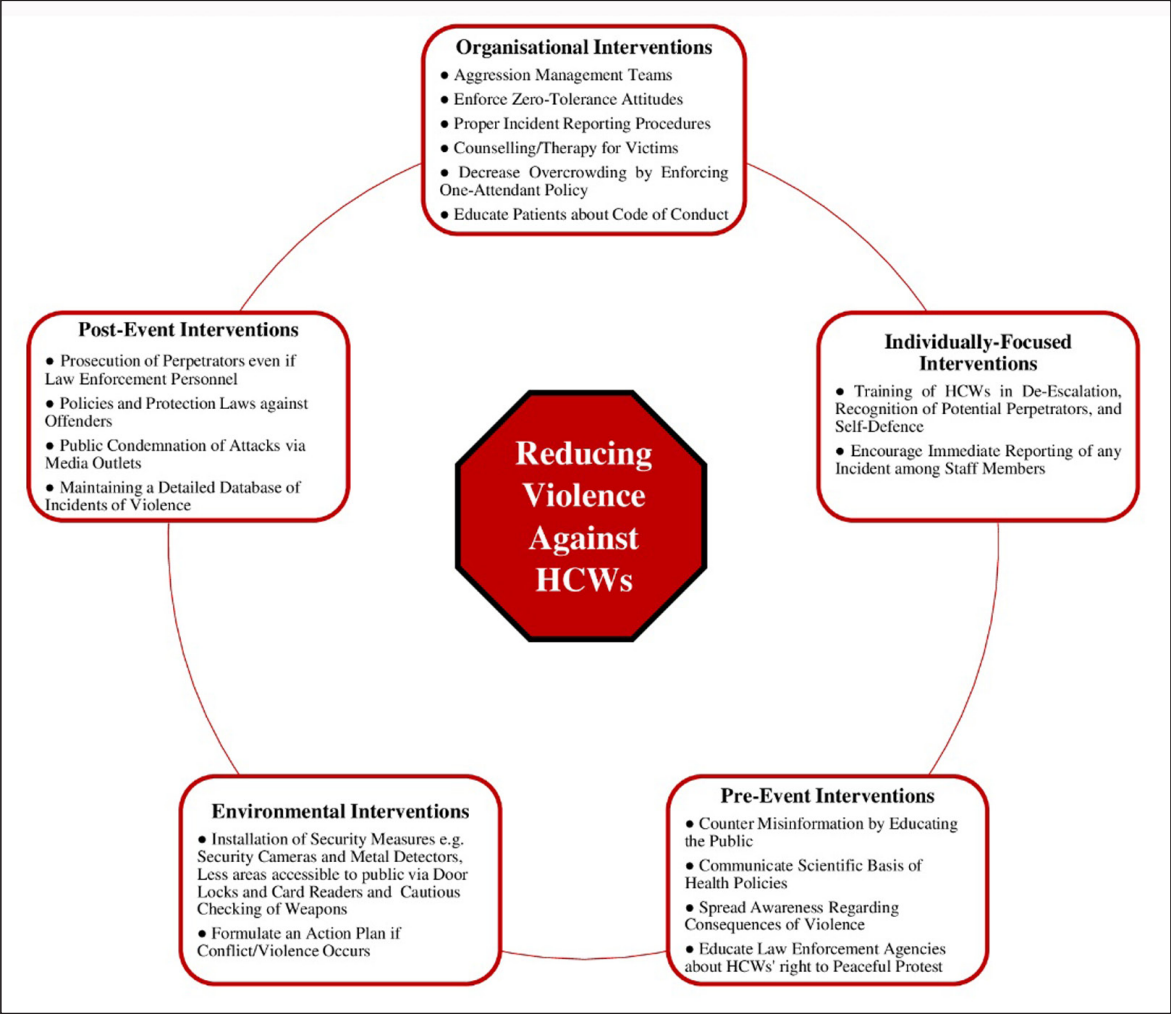
Summarises general measures to reduce violence against HCWs.

## Conclusion

Healthcare professionals are the most valuable asset during a health crisis, and therefore their safety and well-being should be of the utmost priority. The issue of healthcare violence must be acknowledged and addressed at both a national and an international level. The government, health policy makers, media organisations, and community engagement groups, as well as the medical fraternity, must work together to eliminate violence and to ensure work safety for the medical profession.
